# Using and Joining a Franchised Private Sector Provider Network in Myanmar

**DOI:** 10.1371/journal.pone.0028364

**Published:** 2011-12-13

**Authors:** Kathryn O'Connell, Mo Hom, Tin Aung, Marc Theuss, Dale Huntington

**Affiliations:** 1 Population Services International, Nairobi, Kenya; 2 Population Services International/Myanmar, Bahan Township, Yangon, Myanmar; 3 Department of Reproductive Health and Research, World Health Organization, Geneva, Switzerland; Kenya Medical Research Institute - Wellcome Trust Research Programme, Kenya

## Abstract

**Background:**

Quality is central to understanding provider motivations to join and remain within a social franchising network. Quality also appears as a key issue from the client's perspective, and may influence why a client chooses to use a franchised provider over another type of provider. The dynamic relationships between providers of social franchising clinics and clients who use these services have not been thoroughly investigated in the context of Myanmar, which has an established social franchising network. This study examines client motivations to use a Sun Quality Health network provider and provider motivations to join and remain in the Sun Quality Health network. Taken together, these two aims provide an opportunity to explore the symbiotic relationship between client satisfaction and provider incentives to increase the utilization of reproductive health care services.

**Methods and Findings:**

Results from a series of focus group discussions with clients of reproductive health services and franchised providers shows that women chose health services provided by franchised private sector general practitioners because of its perceived higher quality, associated with the availability of effective, affordable, drugs. A key finding of the study is associated with providers. Provider focus group discussions indicate that a principle determinate for joining and remaining in the Sun Quality Health Network was serving the poor.

## Introduction

With a population of approximately 55 million people, Myanmar is one of the poorest countries in Asia and is classified by the United Nations as one of the least developed countries [1]. The maternal mortality rate in Myanmar is estimated at 140 per 100,000 live births for urban areas and 316 per 100,000 live births in rural areas. The contraceptive prevalence rate among married women is 33% and the unmet need for contraception among married women is estimated to be 19.1% [2].

Sexual or reproductive health services in Myanmar have only been available through the public sector since 1991, and these services are restricted. Services include maternity care (antenatal, delivery and post natal services); abortions; prevention or treatment of sexually transmitted diseases, including condom promotion programmes; and family planning [3]. For example, in Myanmar, government clinics only provide contraception to married women, and are only available in 40% of the country. In the remaining 193 townships, the public sector offers no family planning services [4]. Data also show that among currently married women using a contraceptive method, 51.8% received their modern method through the private sector and 42% through the public sector. The majority of these women received this from a public sector hospital (24.7%) followed by a drug store (13.1%) (Ministry of Immigration and Population, 2009).

### Social Franchising Services

Engaging the private sector in health care delivery can help to expand access to health and social services among low-income households, particularly in situations where public health facilities are limited in their geographical scope and service provision. In particular, social franchising has been used as a mechanism to reach low income populations through the private sector.

Franchising is a business model that has been defined as ‘an arrangement whereby a manufacturer or marketer of a product or service (the franchiser) grants exclusive rights to local independent entrepreneurs (franchises) to conduct business in a prescribed manner in a certain place of a specified period [5]–[7]. Examples of these services can be found in Mexico, India, Pakistan, Cambodia, Ethiopia, and Kenya and not only include sexual and reproductive services, but also TB, pneumonia and child survival. In India, the franchised network called Janani provides contraceptives at reduced prices through contraceptive shops in 1996. As a result there are currently more than 11,000 health providers participating in the Janani network [8]. In Ethiopia the Biruh Tesfa, or ‘Ray of Hope’ franchised network provide family planning and reproductive health services, and commenced in 2000. Members are existing community-based health care providers, including community health agents, birth attendants, market place providers and workplace providers [9].

Social franchising programmes typically include sexual and reproductive health services, but not exclusively, e.g., tuberculosis, malaria are also often included in the franchised programme. The growth in social franchising can be viewed as an evolution of the contraceptive social marketing programmes and hence will most commonly always include a family planning element. The development and expansion of social franchise programmes can be viewed as a positive sign of public-private sector partnership and is not suggestive of public sector failure. The franchisees (i.e., those providers who join the franchise) are in fact for-profit providers of health care services. They will provide a full range of primary health care services, both curative and preventive, seeking payment using locally determined means (either third party of direct use fees). For those services and commodities that are supported by the franchise programme the providers accept limitations of fees that can be charged (e.g., caps are placed on the amount that can be charged for contraceptive methods).

Many questions remain unanswered as to why women will choose to use a franchised provider over another type of health care service in settings where multiple provider types exist. While some research has suggested that clients typically choose franchised providers based on their perceptions of service quality, lower prices as a result of subsidized commodities, provider knowledge and preferences for services with different characteristics [10]–[11] evidence from other studies conducted in different countries has provided mixed results. For example, in a study using cross sectional data [9] examined the associations between franchise membership and family planning and reproductive health outcomes for both the member provider and the client in three settings. Results showed that franchising had a positive affect with both general and family planning volumes, and the number of family planning brands available. However, only in Pakistan was attendance at a franchised health outlet significantly associated with reporting an intention to return to the same health establishment – and in Ethiopia, clients were less likely to report an intention to return to the service.

Provider motivations to join a social franchise network are not well understood either. Some evidence suggests that providers may be motivated to become part of a franchise for both monetary and non-monetary reasons: an increase in client volume may convince providers of the usefulness of network participation [12]; opportunities for training [13], [8] and providers' desire to interact with professionals in the same field may also be important motivating factors [6]. Providers may be motivated to join a franchise network because of the perceived social value of the services supported by the franchise program (e.g. family planning and subsidized health care for the less privileged) [9]. Other non-financial influences may also drive private providers to join a social franchised network, such as improving their reputation [14] and greater local responsibility [6].

In Myanmar, where a mature social franchising program has existed since 1999, provides an opportunity to address why women will choose to use a franchised provider over another type of health care service in settings where multiple provider types exist. It also provides a chance to further understand provider motivations to join and then stay within a clinic.

### Setting

Population Services International/Myanmar (PSI/Myanmar) is a non-profit, non-political and non-religious organization that uses social franchising and social marketing programs to empower low-income and vulnerable people to lead healthier lives. PSI/Myanmar is both a Myanmar organization and an affiliate of Population Services International, an international social marketing organization. PSI/Myanmar's programmes include HIV and AIDS, sexual and reproductive health, malaria, tuberculosis, pneumonia and diarrhoeal disease services.

Social marketing, as practiced by PSI, combines education to motivate healthy behaviour with the provision of needed health products and services to lower-income persons. PSI motivates a wide variety of healthy behaviours, including use of products and services. Social franchising through PSI/Myanmar involves engaging private medical practitioners to add new services to the range of services they already offer, attracting them with training, technical support, subsidized goods, free advertising, links to other providers, and to a brand that represents quality, accessibility, and affordability.

PSI/Myanmar's Sun Quality Health network is a franchise network of licensed general practitioners launched by PSI/Myanmar in 2001. Sun Quality Health members are primary health-care providers that offer a range of health care services (including birth spacing, and sexual and reproductive health treatment). The clinics are generally located in urban and peri-urban areas and small towns.

Since its launch, the network has expanded considerably in both scale and types of services it supports. Approximately 120 new providers join the network each year through induction training courses on birth spacing, adolescent reproductive health, management and voluntary counselling and testing services. Since the first group of providers was enrolled in the network, a total of 1,173 providers have participated in the induction training and 1,006 remained active in 2009.

During induction training of each disease area including sexual and reproductive health, providers are given a 2–3 day training course; a multitude of informational materials, promotional Sun Quality Health H products; and access to branded, high-quality products. For sexual and reproductive health issues, the training covers birth spacing, adolescent reproductive health, management of sexually transmitted infections, and voluntary counselling and testing services. The distributed products include contraceptives, other drugs and medical products. All of these products are to be provided either free of charge or at highly subsidized prices to their patients. In return, the providers agree to keep clinical records, to respect service standards and to adhere to a price structure on Sun Quality Health provided contraceptives, drugs and other medical products. Quality of care standards are monitored through monthly monitoring visits using standardized check-lists for each disease area including sexual and reproductive health (e.g. infection prevention measures, stock status, price, record keeping, and reporting).

### Objectives

This study examines client motivations to use a Sun Quality Health network provider and provider motivations to join and remain in the network. Three research questions guided the study: What are clients' attitudes towards the quality of sexual and reproductive health services in the private health sector? To what extent do clients perceive that sexual and reproductive services from social franchising services adhere to these quality standards? What are the provider incentives to join and remain within a social franchising network in Myanmar?

## Methods

### Target groups

To understand the complexity of factors contributing to client expectations, this qualitative study included three different target groups. This first target group comprised of married female clients aged 15–49, receiving reproductive health care from franchised Sun Quality Health [SQH] clinics or non Sun Quality Health franchised [non-SQH] clinics. For inclusion into the survey, these women also had at least one child and were currently using a modern family planning method. The second target group included female sex workers, who had sought care for sexually transmitted infections care from franchised and non franchised clinics [SQH and non-SQH]. The third target group included male and female SQH clinic providers.

Women of reproductive age, using a variety of services from SQH and non SQH clinics, helped to understand client expectations. Because sexually transmitted infections are stigmatized in the Myanmar context, particular efforts were made by the research team to identify women in appropriate contexts to explore the opinions and views of women using private clinics for STI problems. To understand the provider incentives to join and remain in the network, the providers were recruited from clinics with a diverse range of client load, service provision, and length of SQH membership. In each group, providers with high case load in different health areas were recruited in order to obtain a holistic view of the different services provided by the SQH network. Most of the providers recruited for focus group discussions served about 300 clients per month; some have high family planning clients while some have more tuberculosis, pneumonia or malaria patients. Moreover, providers with 3–5 years of SQH memberships were also purposively selected to understand both motivations for joining the network and reasons for remaining in the network.

### Procedures

The following three methods were used to recruit women for the study according to the target group:. (i) To recruit SQH participants, the research team made contact with the SQH providers from clinics located in six different peri-urban areas of Yangon and the Bago Divisions. SQH providers were informed of the study and permission was obtained to recruit potential focus group respondents directly from the clinic. Researchers then screened respondents directly at the clinic, after receiving consultations from providers; (ii) o recruit other women who used non-SQH franchised clinics, a snowball method was used. Women who were approached at SQH clinics were asked if they knew other women like themselves who might be interested in participating in the study. Researchers accompanied SQH participants to the houses of potential respondents. Non-SQH participants were then screened and informed of the study in their homes; (iii) To recruit female sex workers, researchers approached two non-governmental clinics that served as drop-in centres for this target group. These centres provided services for sexually transmitted diseases and provided a suitable environment in which to approach this target group for participation in the study. Female sex workers were recruited in a similar manner to respondents attending SQH clinics, with permission being obtained first from the clinic provider and female sex-workers recruited on site of the clinic. To recruit SQH clinic providers, providers were purposely selected for inclusion into the survey from twelve different clinics located in two townships of Myanmar, one township from plain region and one from mountainous region. Six providers from each township were approached directly by telephone 2–3 days prior to the discussion and provided the overview of the study including study background and objectives. Providers who agreed to participate in the study were then informed about the date, time and place of the focus group discussion. A screening document was used to ensure that the criteria and targets for group allocation were met.

Focus group discussions were conducted in November 2009, with around 6–8 participants per group. Focus group discussions with women of reproductive age were conducted in peri-urban areas: either in a rented, private room or at the house of a participant. FGDs with female sex workers were conducted at the drop-in centres. Focus group discussions with providers were conducted in urban areas, in rented, private rooms. On arrival to the focus group discussion, participants were introduced to the moderator and to each other. An introduction was then given by the moderator, which outlined the project, the aims, and specifically, the task of the focus group. On completion of the discussion, quick summaries of the findings were fed back to the group. All discussions were held in private rooms and tape recorded for accuracy. Recordings from all interviews were transcribed verbatim into Burmese.

Informed, written consent was obtained from all participants in the study prior to the conduct of the focus group discussions. All participants were provided with an information sheet, describing the objectives of the study and measures to ensure confidentiality.

Interview guides for clients (married women and female sex workers) used open-ended questions to address several key topics related to women's experiences with providers at the different clinics. A participatory mapping exercise was used to identify private practitioners in the area where women lived, and asked which providers they went to for different issues, including family planning, pre and post natal care and sexually transmitted infections. Prompts were then used to address issues such as satisfaction with the practitioner, provider skills, the relative importance of different services as well as positive or negative experiences with different types of providers.

Interview guides with providers addressed a number of topics related to their motivations for joining and remaining with the SQH clinics. A lose narrative and discursive framework was used to illicit information from providers.

Ethical approval for this study was obtained through a two stage review process. A locally convened ethical review board (Ethical Review Committee of PSI/Myanmar) in Yangon, Myanmar provided the first stage review. Subsequently the full study protocol, discussion guides, informed consent forms and data collection procedures were reviewed by the Reproductive Health and Research Department, World Health Organization's Scientific and Ethical Review Group. After gaining approval from that stage of review, the study materials were reviewed and approved by the World Health Organization's Ethical Review Committee.

### Analysis

A thematic based analysis was used for this study. Focus group discussions were fully transcribed into Burmese. Each transcript was then read by two researchers. As we had a preconceived set of interest areas in this research, a descriptive list of deductive codes were used as a general guide for the analytic approach. Descriptive codes were then thematically identified and arranged into a smaller set of broader themes which captured the main topics emerging from the data. This analysis reported on the frequency with which the themes were reported in each group was analyzed for patterns. This procedure helped clarify which themes emerge consistently across all groups and which are idiosyncratic to each type of user. Comparisons were made across groups with respect to the emergent themes. Inter-rater reliability was checked a third researcher, who reviewed all of the transcript codes, compared findings and resolved any discrepancies through discussion with the larger team of coders. Upon completion and writing of the results, the results were presented to the data analysis team to confirm the findings and quotes again. Quotes used were then translated and verified a second time with translators. Results presented in this paper present themes that emerged consistently across all groups.

## Results

In total, 12 Focus group discussions were conducted for this survey. Six Focus group discussions were conducted with married women using SQH and non-SQH services.

Four FGDs were conducted with female sex workers, all who were seeking treatment for sexually transmitted infections from private providers or at drop-in-centres. Two focus group discussions were conducted with SQH clinic providers, drawn from two areas of Myanmar and from different clinics. In each FGD, 4 male and 2 female providers participated in the discussion.

### Clinical characteristics or attributes of clinics

Respondents addressed the importance of having access to affordable services for their sexual and reproductive health needs. Both groups of women stressed the importance of being able to receive services from providers that offered lower consultation or service charges as well as less expensive products. However, while respondents discussed the importance of service fees at the clinics, they did not typically make a distinction between the product price and service charge.

While respondents acknowledged that fees and products in the private sector could be high, they did raise the issue that SQH clinic fees were generally less expensive. Respondents also reported how other, private clinics charged more than SQH clinics, and stressed the importance of lower prices as an important attribute of the clinics. Respondents who had attended SQH clinics for reproductive health issues raised that point that SQH clinics provided more affordable fees than other private clinics. The quote below illustrates respondents' perceived affordability of SQH clinics as it compares to other private providers.

*“When it [service fees] costs 250 kyat at other clinics, here [in the SQH clinic] it costs about 150 kyat. One hundred kyat is of great importance for the poor.”*

- *FGD with FSWs from Drop-In Center, Yangon*

The perceived availability of different products, as well as a having an assortment of different types of health products, was also an important attribute of clinics. Having a range of health products, and ensuring these were listed and posted in a visible location at the clinic, was cited as an important feature of private clinics. Respondents suggested that having a range of health products encouraged them to come back to the clinic, as they could review their options for using different types of products and enquire about different types of methods that might be available to them.

*“In “SQH” clinics they have written down the list of products which are available there. From that list, I came to notice that it has a variety of products and therefore I go there.”*

- *FGD with women from peri-urban area, Yangon*

The perception of ‘quality’ products and medicines for sexual and reproductive health services was also revealed as an important attribute of clinics. Respondents discussed concerns surrounding sub-standard or fake medicines that may be sold to them, or products that might be expired. These particular concerns seemed to be related to medicine stalls selling drugs at markets. Respondents who attended SQH clinics were believed to stock ‘better quality’ medicines than other clinics, referring to medicines as ‘safer’, more ‘effective’ and less likely to be expired than medicines stocked with other private providers. They also flagged the importance of receiving a medicine that was not expired, and believed that SQH clinics were far less likely to sell expired drugs.

Privacy was highlighted as an important attribute of clinics for all the respondents. Being able to discuss sexual and reproductive health issues in the confines of a private room, or at least far away enough from where others can hear, was deemed as a very important feature of clinics. Many clients disclosed that they were more likely to be examined and given treatment in privacy at the SQH clinic than other sources of care.

*“I don't like going to that other [non-SQH] doctor since I feel like being in the open space at his clinic. Since I'm doing this work, it is important for me to have a discussion privately and have a family-like relationship between me and the provider.”*

- *FGD with FSWs* from Drop-In Center, Bago

Some women expressed being pleased with the range of different types of providers to discuss sexual and reproductive health issues. That is, having access to providers with different skills, as well as having access to both male and female providers. They also flagged that this was an important attribute that could be found at some of the SQH clinics. In particular, they valued being able to access female doctors and assistants at the clinics:

*“I feel secure (lone-chone) at the time of being examined. There are only three persons in the room: the doctor, assistant and me.”*

- *FGD with FSWs* from Drop-In Center, Yangon

Having access to printed material was another important feature that emerged from the focus group discussions. Many clients stated that they liked to read education pamphlets and posters during waiting times and appreciated the availability of pamphlets that could be taken away. Respondents specifically mentioned how the different types of printed materials that were visible in SQH clinics was being an important feature of the health care facility.

### Personal and professional characteristics of providers

Many clients disclosed that they liked to seek care from providers because they felt that the provider was ‘like a family member’. As a result, they felt comfortable discussing a variety of health issues, especially related to sensitive issues surrounding family planning and STIs. Respondents also mentioned that friendliness and attention were important components that were important in a provider, and affected whether or not they would return to that provider. They mentioned that:

*“Saya (male doctor, at the SQH clinic) is like a family member so I can talk openly with him. There is another clinic in my area which is opened by another doctor from somewhere else. Since I don't know him personally, I don't feel comfortable to talk with him.”*

- *FGD with women from peri-urban area, Yangon*

Respondents relied on the providers with whom they had family-like relationships. Respondents also expressed their satisfaction with the ‘friendliness’ of the providers from SQH clinics, lending support that providers from SQH clinics may have some of these attributes.:

*“Sayama (female doctor, at the SQH clinic) is friendly with every patient. She gives about 10–15 minutes to each patient. She examines, explains and asks questions.”*

- FGD with women from peri-urban area, Bago

*“He's like a family member and it is very good to discuss with him. It means I can talk to him openly. He is so nice that I feel as if half of my illness is cured as soon as I see him.*

- *FGD with women from peri-urban area, Yangon*

Having the advice from a friend or family member was a key aspect of the decision-making process in choosing the health care provider. Respondents typically discussed the most appropriate provider to deal with a particular illness through their network acquaintances and contacts. Personal referrals were often an important source of information about a health care provider for many women, and quite often, respondents reported that others had recommended providers from SQH clinics.

*We learn about providers by word of mouth. For example, if this Saya (male doctor) is treating an illness we women may talk about it to each other. We say things like ‘this Saya can handle this kind of illness.”*

- *FGD with women from peri-urban area, Yangon*

Respondents also relied on the providers whom they believed had the experience and skills to cure their illness or disease. For many of them, one of the most important things in choosing a clinic was not only the outcome of the treatment and efficacy of the medicine, but the competency of the providers. Respondents often discussed the competencies of the SQH providers while comparing them to other types of private providers.

*“I do not dare to go to another clinic because there was an accident (because of a wrong treatment) by that provider. With Saya [from SQH clinic], I trust him. I rely on him as well. Many people get used to go to Saya. He really cures our diseases.”*

- *FGD with women from peri-urban area, Yangon*

Some women stated that once they believed in the competence of the provider in treating the patients, they will go to that provider, and not anyone else.

Respondents also valued what they called ‘good-will’ of the providers. A provider with good will ranges from someone who will give treatment for free, a provider that will open their clinic when environmental factors may make this difficult, and a provider that shows caution about the types of medicines that a provider is giving. The context or relevance of goodwill can be illustrated in the quotes below:

*“Sayar is very nice and has “Say-ta-nar (good will).” At times when I don't have enough money in hand to take treatment at Saya's clinic, I still go to see him. When I explain about my condition, he doesn't take any money.”*

- *FGD with FSWs from Drop-In Center, Bago*

### Providers

This section discusses two components that were purposely addressed in the interview guides: what are the provider incentives, both monetary and non-monetary, to join and remain within a social franchising network in Myanmar?

Increased income was rarely stated a provider's primary expectation in joining the SQH network. In fact, providers frequently emphasized that they were worried that their earnings might decrease as a result of joining the network.

*“In the beginning since we were charging for 1,000–1,500 (for injecting) before and now it is only 500 earning decreased. But the client increased from say 5 to 15 or 20 and it come to same income.”*

- male doctor

Although providers reported that earnings might decrease, some of the discussions suggest that they may be experiencing an increase in the volume of patients for other types of services, perhaps alleviating any concerns that they may in fact lose money by joining the network. The fact that they may find an increase in their earnings suggests an important spill-over effect on their overall earnings. Indeed, providers indicated that their initial worries of decreased income didn't materialise because of increase in the volume of patients.

*“We got even more clients. (After the clients came to know about this clinic) they came back to this clinic whenever they had other health problems. So, we got even more repeat visits from existing clients.”*

- male doctor

As suggested above, non-monetary incentives emerged from the focus group discussions as being important considerations for a provider to join and remain a member in the SQH network.

The findings show that while providers recognize and value the monetary (or commercial component) of their private practice, the benefits of reaching the poor and providing affordable medicines were very important considerations in joining the SQH network. Providers articulated a sense of solidarity with the poor people they serve defining themselves as servants to the poor rather than simply service providers for commercial ends. SQH was conceptualised as an international body that supported and strengthened GPs commitment to serve the poor.

*“We could not treat them at a cheap price by ourselves. But when the network evolved I was able to support poor people to a greater extent.”*

- male doctor

Another key advantage in joining the network included an increased self confidence in a provider's clinical skills. Providers believed that their ability to diagnose and treat patients with different illness improved after joining the SQH network. This had the positive outcome of increasing trust, or perhaps confidence, among their clients.

*“Previously, we referred patients to hospital if a patient was suspected of TB. But, we can treat by ourselves now. As we can manage by ourselves, we develop more trust among patients.”*

- male doctor

Related to their sense of improved clinical skills was the finding that they had increased their access to a regular supply to perceivably more efficacious medicines, i.e., the SQH branded products. These medications were perceived as more reliable than those being stocked in open markets or through other distributers.

Providers were extremely enthusiastic to have ‘newer’ medicines and a greater range of these medicines, that were not available to them prior to joining the Sun Quality Health network.

Providers expected to improve their capacities, skills and knowledge as a result of joining the clinic and saw the training courses offered through SQH membership, as an important means to further their skills. Providers also described progression in the development of their skills in diagnosis as well as treatment of various illnesses as a result of joining and being a member of the network. Related to this, professional networking and participating in a nation-wide organization were key considerations that encouraged providers to join the SQH network.

*“Previously he (another doctor) stayed by himself and I stayed by myself spending time in a tiny clinic. We never had a chance to go outside (and meet each other). It happened like that. We now meet each other at the training.”*

- male doctor

## Discussion

The study results reinforced previous research that showed women chose to access treatment and services from general practitioners working in a private sector franchised network even in settings where multiple types of providers exist. Respondents valued the ‘clinical’ characteristics or attributes of the private clinics. That is, the extent to which services were perceived as affordable, products were available and informational material was displayed. In addition, respondents reported the importance of ensuring privacy and confidentiality when receiving sexual and reproductive health services, and highlighted other significant features of the clinic and medical products such as waiting time, hygiene, and comfort. The investments made by the social franchise programme in signage, infrastructure improvement and predictable supplies of essential reproductive health commodities are clearly elements of the perceived quality that women in this study valued.

Findings show that respondents valued the personal and professional characteristics of providers, in addition to the availability of the services and other features of the clinic and medical products. This study revealed that importance attached to social factors such as familiarity, trust, competency, reputation, age, gender, friendliness and respectfulness of providers. For some attributes, these components appeared to affect the clients' preferences in using a SQH clinic as well as returning to that clinic. Women's perceptions of the physician's personal (as opposed to professional) characteristics were also shown to be important considerations in the study's findings. For example, the sense of a trusting relationship with the provider who was viewed as being a member of the local community was expressed by many of the women who took part in the focus group discussions. This has implications for the recruitment and sustained membership of providers in a social franchise

Insights from the provider study contradict a body of literature which suggests that financial benefit is a key determinant of joining, and remaining within, a franchised network. Statements from these private practice, fee-for-service general practitioners suggest a profound sense of social responsibility as the primary motivation for joining and remaining in the social franchise. Although every provider who took part in the study operates an essentially commercial enterprise, their willingness to provide low or no-profit services as a way of giving-back to the communities speaks directly to critiques of the private health care sector as being economically predatory. It may be a function of selection bias (i.e., those providers who chose to join a social franchise are more inclined to have a social responsibility ethic), so we are not generalizing to the entire private sector market. Although important spill-over effects were suggested (e.g., increased patient volumes, treatment for other aliments that are not included in the capitated fee structure of Sun Quality Health franchise), a key determinate for joining and remaining in the Sun Quality Health franchise as expressed by the franchised providers was serving the poor.

The physicians who joined Sun Quality Health franchise also expressed strong interest in deriving benefit from participating in the network – in addition to giving to their communities. The possibility of continuing medical education, and professional dialogue was highly valued. These critical network functions contribute directly to creating conditions for self-regulation and oversight controls that can be exercised by the franchisor.

The experience of the Sun Quality Health network in Myanmar point to several areas where this social franchise network is making an important contributions to national health care programs. By increasing access to affordable contraceptives, drugs and products, social franchises are contributing to the goal of universal coverage of reproductive health services. The networks are creating conditions that are highly conducive to professional development among their member physicians, enacting soft-regulation wherein the network managers provide essential clinical oversight. The findings from this show that quality of care and equitable access is a central consideration to both patients and providers in the private sector.
